# Osmotic Effects Induced by Pore-Forming Agent Nystatin: From Lipid Vesicles to the Cell

**DOI:** 10.1371/journal.pone.0165098

**Published:** 2016-10-27

**Authors:** Špela Zemljič Jokhadar, Bojan Božič, Luka Kristanc, Gregor Gomišček

**Affiliations:** 1 Institute of Biophysics, Faculty of Medicine, University of Ljubljana, Ljubljana, Slovenia; 2 Faculty of Health Sciences, University of Ljubljana, Ljubljana, Slovenia; Nagoya University, JAPAN

## Abstract

The responses of Chinese hamster ovary epithelial cells, caused by the pore-forming agent nystatin, were investigated using brightfield and fluorescence microscopy. Different phenomena, i.e., the detachment of cells, the formation of blebs, the occurrence of “cell-vesicles” and cell ruptures, were observed. These phenomena were compared to those discovered in giant lipid vesicles. A theoretical model, based on the osmotic effects that occur due to the size-discriminating nystatin transmembrane pores in lipid vesicles, was extended with a term that considers the conservation of the electric charge density in order to describe the cell’s behavior. The increase of the cellular volume was predicted and correlated with the observed phenomena.

## Introduction

The effects of antibiotics on cell membranes have always been the subject of wide-ranging investigations. Polyene antibiotics like amphotericin B and nystatin belong to a class of biologically active bacterial metabolites, which are most commonly used to treat fungal infections in humans due to their higher affinity for ergosterol than for cholesterol [1,2]. The research on polyenes has become increasingly important as a result of the higher incidence of systemic fungal infections, especially with the increasing prevalence of immunocompromised persons [3]. Recently, new lipid formulations of nystatin with a lower toxicity and better water solubility were developed, which is particularly important because nystatin is active against a broad spectrum of fungal pathogens [4].

The main biological activity of the pore-forming agents seems to result from their amphipathic structure [5], which enables the formation of barrel-like, membrane-spanning channels in the plasma membrane [6,7]. These transmembrane pores, with their effective radii that are comparable to the size of small molecules, have size-selective properties [8–10]. They increase the plasma membrane permeability, especially for ions and small molecules, which causes a disturbance in cellular electrochemical gradients and ultimately leads to cell lysis [1]. The different properties of the pore-forming agents have been widely investigated. These studies were devoted primarily to the pore-formation process, i.e., their membrane binding, partitioning and self-aggregation [11,12], and secondly to the physiologic implications in the case of different cell types. The studies of the nystatin and amphotericin B activity have demonstrated the suppression of growth and the death of fungal and leishmanial cells [13–15], while in various mammalian cells morphological responses and cellular ion concentration changes were found [16–19]. Nystatin has been used in experiments investigating the electrical properties of different tight epithelia, such as mammalian urinary bladder and colon epithelia, which characterized the conductances of the nystatin transmembrane pores for Na^+^, K^+^ and Cl^-^ [20,21]. In addition, it was observed that nystatin influences many mammalian cellular functions, among others the different intracellular signaling processes induced through the caveolae-associated proteins [22,23].

Since different lipid bilayers constitute around 40% of biological membranes, the pore-formation process has been extensively studied using different lipid model membranes, especially lipid vesicles with diameters below 1 μm [2,24,25]. In these studies, the relatively simple composition and the closed membrane surface of the vesicles enable investigations of the pore-formation processes based on leakage experiments conducted on a large number of vesicles. Studies of the effects of nystatin on lipid bilayers have also recently been undertaken on giant lipid vesicles (GUVs), the sizes of which are comparable to the sizes of the cells. These experiments, which make possible observations of single vesicles, have offered some new insights into the pore-formation process [26]. They revealed a variety of phenomena, i.e., vesicle shape changes and various osmotic phenomena, such as the formation of transient tension pores and vesicle ruptures. In addition, a theoretical model based on the theory of osmotic lysis [27] and the pore-diffusion theory [28] was developed in order to understand the basic experimental results obtained from GUVs with different membrane compositions [29].

A straightforward question arises as to how the results obtained from GUVs can be correlated with the effects of nystatin on the cells. In this work we focus on the characteristic responses of Chinese hamster ovary (CHO) epithelial cells at different nystatin concentrations. We also extend the theoretical model applied in GUVs to take into consideration the conservation of the electric charge density in cells. The model describes the increase of the cellular volume induced by the passages of most prevalent ions through the nystatin pores and predicts different types of cell membrane behavior. Finally, the results of the experiments on the CHO cells and the theoretical predictions are discussed in relation to the findings obtained with GUVs to gain new insights into the pore-formation process and its influences on the mammalian cell.

## Materials and Methods

### Preparation of the CHO cells

The Chinese hamster ovary (CHO) epithelial cells (ECAC) were grown in Serum Reduced MEM medium (Gibco, USA) supplemented with antibiotics (penicillin-streptomycin; Gibco, USA), L-glutamine (2 mmol/l) and 5% fetal bovine serum (Gibco, USA) at 37°C in a CO_2_ incubator (5% CO_2_, 95% relative humidity). For the experiments the cells were detached with TrypleTMexpress (Life Technologies, USA) and seeded (5 × 10^4^ cells/ml) to fibronectin-coated Delta T Dish with a 0.17-mm-thick glass bottom (Bioptechs Inc., USA). The experiments were performed one day after the cells were plated. Prior to the measurements, the Serum Reduced MEM was substituted with Leibovitz’s L-15 medium (Gibco, USA) supplemented with 10% FBS.

For the leakage experiments the Calcein AM solution (Life Technologies, USA) was added to the cells in the growth medium (dilution 1:100) one day after plating. The cells were incubated for 20 min. Afterwards, they were washed and transferred to the microscope stage where the experiments were performed. To observe the morphological changes of the cell and the reorganization of the actin cytoskeleton caused by the nystatin in living cells, the cells that expressed GFP-tagged actin were prepared. One day after plating the transfection reagent Metafectene Pro (Biontex, Germany) was used to incorporate the p^CAG^-LifeAct-TagGFP2 (Ibidi, Germany) plasmid into the cells. After 24 hours of incubation the experiments were performed.

### Experimental set-up and procedure

The cells were studied using optical microscopy with brightfield and fluorescence techniques. They were observed with a Nikon ECLIPSE TE2000-E microscope (Plan Apo TIRF objective, magnification 60×, NA = 1.45). Using the brightfield and epi-fluorescence techniques (high-intensity light source: Hg 100 W, excitation filter: EX510-560, absorption filter: BA590), the images were captured with a digital camera (DS-2M BW, Nikon, Japan). For the confocal microscopy a Nikon C1 system was used (light source: xenon argon laser 488, excitation filter: EX510-560, absorption filter: BA590). The images of the cells were recorded with the same field of view for 60 min, at a rate of 1 frame per second in brightfield mode and at a rate of 1 frame per 5–10 min in fluorescence microscopy to avoid photo-bleaching. The cells were observed at 37°C in a Delta T Dish, equipped with a Delta T Stage Adapter and a Delta T5^™^ μ-environmental culture-dish controller (Bioptechs Inc., USA).

The nystatin concentrations were 10, 50, 100, 150, 200, 250, 300, 400 and 600 μmol/L. The desired nystatin concentrations were prepared by diluting appropriate volumes of nystatin stock suspension in the Leibovitz’s L-15 medium 3 hours prior to the measurement. The stock suspension of 10 mmol/L nystatin was prepared from the lyophilized nystatin (Fluka, Sigma, USA) prior to the experiment, with pure methanol being used as a solvent.

### Analysis of images

The images were analyzed on the qualitative and quantitative levels. Qualitatively, the characteristic cell behavior caused by the nystatin treatment was determined mainly by a continuous observation of the brightfield images by three independent observers. The actin cortex reorganization and the leakage of the cell content were studied using fluorescence images.

For the quantitative analysis, the measurements of the cell footprint (adhered area) were performed on brightfield images. The cell volume and the volume of the “cell-vesicles” were determined by the addition of consecutive slices of the cell obtained from the confocal microscopy. For each slice in the stack the cell was manually delineated from its surroundings and the obtained cell area was multiplied by the step size that was equal to 0.5 μm. An error of approximately 5% was estimated for the cell volume determination by repeated measurements of the same cells. All the images were analyzed with Nis-Elements AR 3.2 software (Nikon, Japan).

### Control measurements

Since the nystatin suspensions were prepared with pure methanol as a solvent, the control measurements solely with methanol maintained at volume fractions corresponding the nystatin concentrations between 100 and 1000 μmol/L were performed. The methanol volume fraction of 1% corresponds to 100 μmol/L nystatin concentration. The cells were studied at 1, 3, 4, 6 and 10% methanol volume fractions using brightfield optical microscopy, while the fluorescence techniques were used at 3 and 6%. The cells were treated in the same way, incubated for the same period of time, and viewed and analyzed in the same manner as the nystatin-treated cells.

### Viability test

The cell viability was determined using the MTS (= 3-(4,5-dimethylthiazol-2-yl)-5-(3-carboxymethoxyphenyl)-2-(4-sulfophenyl)-2*H*-tetrazolium) test. The cells were plated in 96-well microtiter plates (100 μL, TPP, Switzerland) at a concentration of 5000 cells/well one day before the treatment. The cells were treated for 10, 20, 30, 40, 50 or 60 min by nystatin suspension at concentrations of 100, 150, 200 and 300 μmol/L. Afterwards, the suspension was removed and 100 μL of fresh medium was added to the cell culture in each well, followed by the addition of 20 μL of MTS (CellTiter 96 AQueous Reagent, Promega, USA). After 1 hour the absorbance was measured at 490 nm using a Bio-Tek microplate reader (Bio-Tek Instruments Inc., USA). The absorption corresponds to the amount of the formed soluble formazan product, which is directly proportional to the number of viable cells. For control measurements the cells were treated for 10, 30 or 60 min only by methanol at volume fractions corresponding to nystatin concentrations of 100, 300 and 600 μmol/L.

The cell viability was determined as the ratio between the absorbance measured in the cells treated by nystatin (or by methanol only) and untreated cells. It is expressed as a percentage of the viable cells. The results were obtained from three independent experiments. Eight samples were measured for individual concentration and time point in each experiment.

## Experimental Results

**Fig 1:**
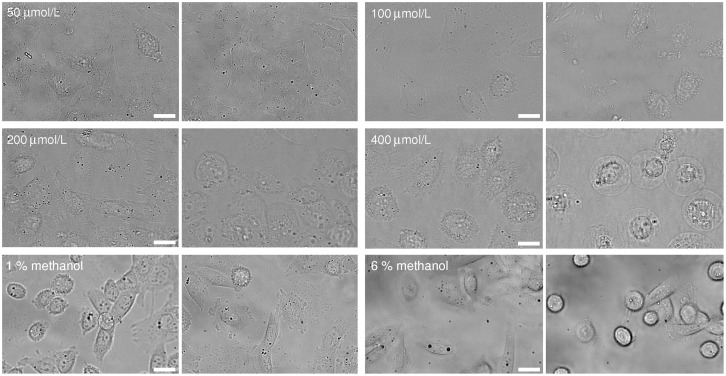
Images of CHO cells observed using brightfield microscopy, sorted by nystatin concentration. The left-hand-side images present the cells 30 s after the addition of nystatin and the right-hand-side images 1 hour after its addition (first two rows). Images for a given concentration are presented in the same field of view. As a control, the images of CHO cells 30 s and 1 hour after the “methanol only” treatment at 1 and 6% methanol (third row). The white bars represent 20 μm.

The characteristic cell behavior changed significantly as the nystatin concentrations increased. At the lowest nystatin concentrations (concentrations up to 50 μmol/L) no significant shape and size changes were observed over a period of 1 hour in comparison to the control experiments (Fig 1). The cell footprint (adhered area) and its height remained unchanged.

At nystatin concentrations between 50 and 150 μmol/L, the cells started to change their shapes (Fig 1). The cells became less flat and their footprints reduced. The latter shrank towards its center due to the cell’s detachment from the surface on its periphery. Average cell footprint decreases between 3% and 13% were experienced at nystatin concentrations of 100 μmol/L and 150 μmol/L. Tethers were observed in certain directions after the footprint shrinkage as remnants of the attached cell membrane. In addition, the blebs whose radius increased significantly during the observation time were observed. At concentrations approaching 150 μmol/L the average bleb radius increased from 0.8 μm to 2.2 μm within the first 10 minutes of the observation time. The blebs appear and disappear at different regions of the cell membrane. The merging of the neighboring blebs was often observed.

At nystatin concentrations around 150 μmol/L the merging of all blebs was detected in most cells and almost spherical, vesicle-like shapes—forms referred to as “cell-vesicles”–were observed. Their average radius was determined to be approximately 13 μm at the end of the observation time. At concentrations between 150 and 300 μmol/L the same phenomena, including the appearance of “cell-vesicles”, were detected, however, the time scale was shorter.

**Fig 2:**
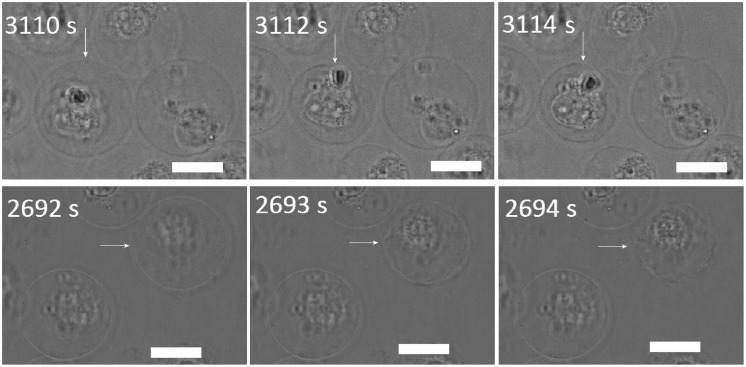
Slow (upper row) and fast (lower row) “cell-vesicle” ruptures as observed by the brightfield microscopy at a 400 μmol/L nystatin concentration. The left-hand-side images represent the “cell-vesicles” right before the rupture, the middle ones during the rupture and the right ones after the rupture. The times after the nystatin addition are indicated on the images. The white bars represent 20 μm.

At nystatin concentrations above 300 μmol/L, an earlier formation of “cell-vesicles” (approximately 5 min after the nystatin addition) compared to the lower nystatin concentrations was observed. At these concentrations, the “cell-vesicle” ruptures were observed for the first time. Slow and fast “cell-vesicle” ruptures could be detected. The slow ruptures are characterized by a significant leakage of the vesicle content and a substantial decrease of the “cell-vesicle” volume in a few seconds (S1 Video). However, the cell membrane reseals afterwards and a “cell-vesicle” with a smaller radius is observed (Fig 2). Fast ruptures, on the other hand, are characterized by a collapse of the “cell-vesicle” within less than a second, accompanied by a total fragmentation of its membrane (Fig 2; S2 Video). The incidence of both ruptures is found to be comparable for the nystatin concentrations examined (between 300 and 600 μmol/L); however, their time of incidence was shorter with an increasing nystatin concentration.

**Fig 3:**
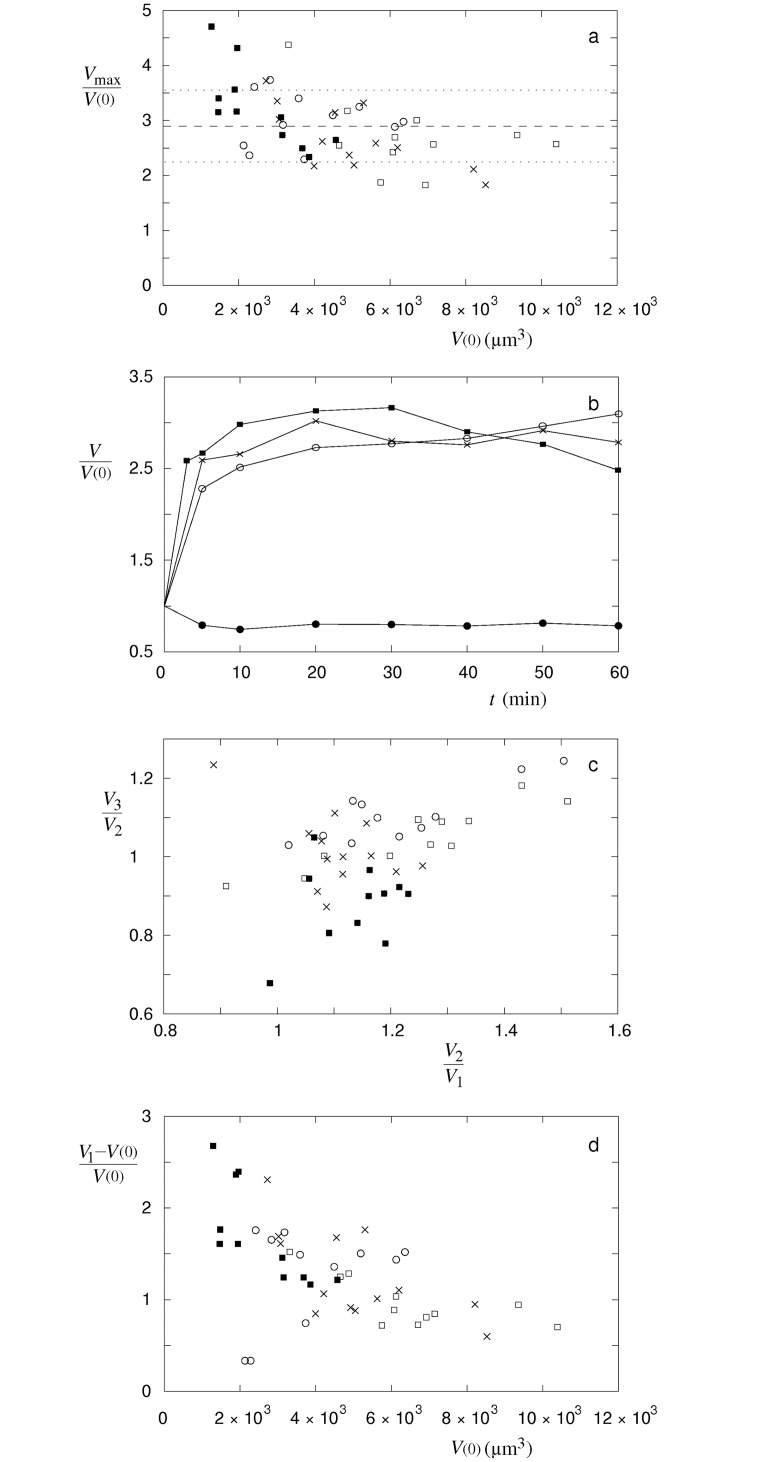
Maximal volumes of single cells, characteristic time behavior patterns of the cell volume changes and ratios between the cell volumes at different phases of the measurement. (a) Maximal volumes of single cells (*V*_max_) relative to their volumes before the treatment (*V*(0)) at 150 (○, n = 11), 300 (□, n = 11), 400 (×, n = 13) and 600 (■, n = 11) μmol/L nystatin concentration. Average value (dashed line) of single cell measurements and standard deviation (dotted lines) are shown. (b) Three characteristic time behavior patterns of volume changes in cells treated by nystatin: “type A” (○), “type B” (×) and “type C” (■). The patterns for single cells with their maximum values closest to 2.9 are presented. As a control, a characteristic behaviour of “methanol only” treated cell at 6% volume fraction is shown (●). The lines are drawn only as a guide to the eye. (c) Ratios between the cell volume at the end of the measurement and in its middle phase (*V*_3_/*V*_2_) versus ratios between the cell volumes in the middle and in the beginning phase of the measurement (*V*_2_/*V*_1_) for the same cells as in (a). The volumes at different phases are defined through the expressions *V*_1_ = [2*V*(5 min) + *V*(10 min)]/3, *V*_2_ = [*V*(20 min) + 2*V*(30 min) + *V*(40 min)]/4 and *V*_3_ = [*V*(50 min) + 2*V*(60 min)]/3. (d) Ratios between the increases of single cell volumes in the beginning of the measurement and their volumes before the treatment ((*V*_1_-*V(0)*)/*V(0)*) versus their volumes before the treatment for the same cells as in (a).

The maximal volumes of single cells relative to their volumes before the treatment do not depend significantly on their volumes (Fig 3a). Similarly, the maximal volume increases of single cells do not differ significantly for 150, 300, 400 and 600 μmol/L nystatin concentration, and an average cell volume increase due to the nystatin application of 2.9 (SD = 0.6) is obtained. In contrast, three characteristic time behavior patterns of the cell volume changes that are dependent on the applied nystatin concentrations were detected (Fig 3b). “Type A” is characterized by an asymptotic increase of the cell volume to the maximal value in the measuring period. The “type B” is characterized by a fast increase of the cell volume which attains its maximal value before the end of the measurement, followed by the volume values which stay around the maximal value. At the “type C” a fast cell volume increase up to the maximal cell volume value is observed in even shorter times, however, the cell volume diminishes afterwards.

The “type A” and “type B” behavior patterns were predominantly detected at 150 and 300 μmol/L nystatin concentrations, the “type B” at 400 μmol/L nystatin concentration and the “type C” at 600 μmol/L nystatin concentration. It has to be mentioned that no “type A” was detected at 600 μmol/L nystatin concentration and no “type C” at 150 μmol/L nystatin concentration. This is demonstrated in Fig 3c, where the characteristic changes in the cell volume between the middle and the beginning phase (*V*_2_/*V*_1_) and between the end and the middle phase (*V*_3_/*V*_2_) of the measurement are depicted for single cells. All types of behavior patterns demonstrate the ratios *V*_2_/*V*_1_ higher than 1 as a consequence of the cell volume increase in the first half of the measuring period whereas the “type A” experiences higher values than the “type B” and the “type C”. However, the ratios of the cell volume changes in the second half of the measuring period (*V*_3_/*V*_2_) are significantly higher than 1 only in the cells characterized by the “type A” behavior pattern as a consequence of a continuous volume increase over the whole measuring period. For the “type B” behavior pattern these ratios are close to 1 due to a fairly constant volume in the second half of the measuring period. For the “type C” behavior pattern the ratio *V*_3_/*V*_2_ is lower than 1 since it is characterized by the cell volume decrease in the second phase of the measurement. Furthermore, the ratios between the increases of single cell volumes in the beginning of the measurement and their volumes before the treatment ((*V*_1_-*V(0)*)/*V(0)*) demonstrate a decrease of their values with an increasing cell volume at 300, 400 and 600 μmol/L nystatin concentration (Fig 3d). The slopes of their linear fits with approximate values equal to– 0.95 × 10^−4^ /μm^3^ (correlation coefficient (R) = — 0.71) for the cell volume increases in a short time at 300 μmol/L nystatin concentration,— 1.80 × 10^−4^ /μm^3^ (R = – 0.66) at 400 μmol/L and– 3.58 × 10^−4^ /μm^3^ (R = — 0.75) at 600 μmol/L, respectively. The slope with an approximate value of 1.34 × 10^−4^ /μm^3^ (R = 0.38) was determined at 150 μmol/L nystatin concentration.

**Fig 4:**
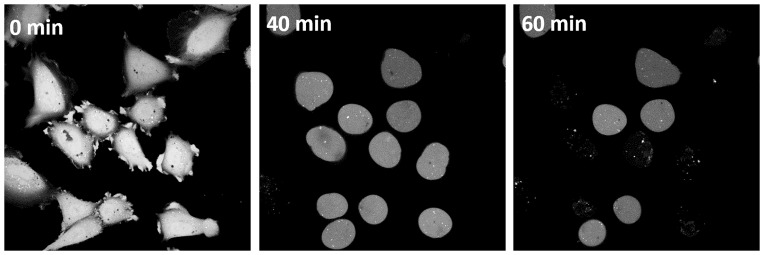
Sample images of calcein-loaded cells treated with 200 μmol/L nystatin. Images of calcein-loaded cells treated with 200 μmol/L nystatin. The images were taken using confocal microscopy at different time points after the nystatin addition (indicated on the images).

Cells marked with fluorescent calcein showed no signal loss during the whole observation time at nystatin concentrations up to 150 μmol/L, in spite of the occurrence of “cell-vesicles”. In the calcein-loaded cells the first disappearance of the fluorescence signal was found in some cells after approximately 40 min at 200 and 250 μmol/L nystatin concentrations (Fig 4). The time of the signal’s disappearance depended on the nystatin concentration: at 300 μmol/L the signal disappeared for the first time in some cells after 35 min, while at 600 μmol/L this occurred already after 25 min. At a 600 μmol/L nystatin concentration, all the cells lost their fluorescent signal during the observation period of 60 min. Furthermore, it should be mentioned that the disappearance of the calcein signal always occurred before the onset of the cell rupture.

**Fig 5:**
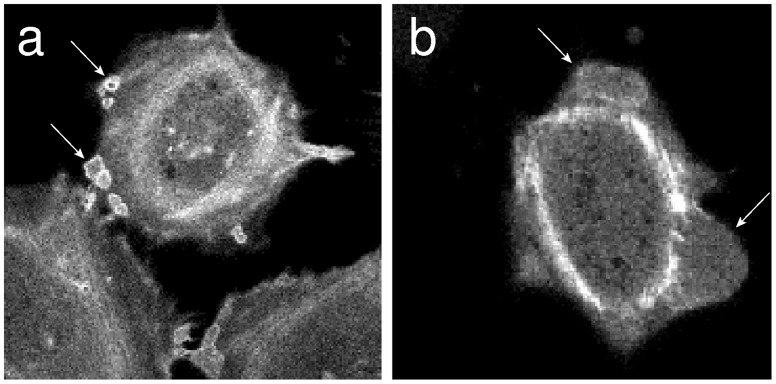
Organization of the actin in blebs. In “living” cells the actin is organized in small (a) and bigger blebs (b), as depicted by fluorescent signal. Some representative blebs are indicated by arrows.

**Fig 6:**
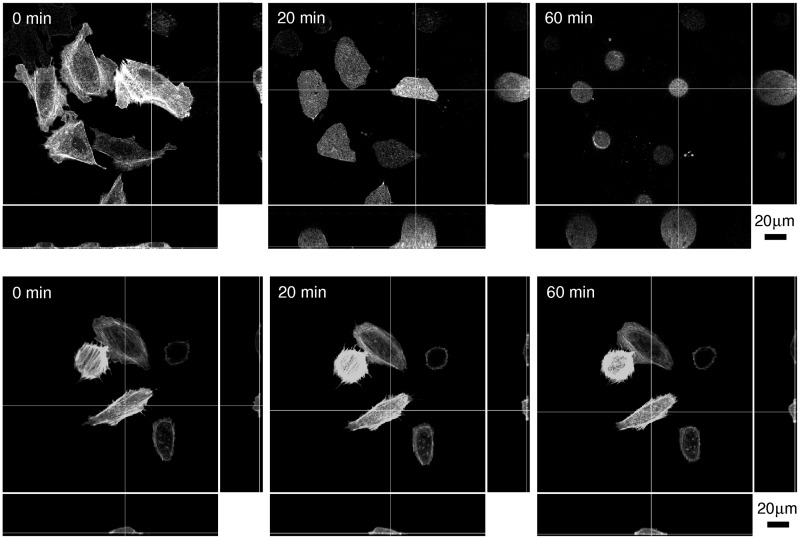
Organization of fluorescent actin structures in “living” cells as seen using confocal microscopy after the addition of 300 μmol/L of nystatin solution. Top view (in the middle) and side views (bottom and right) of the nystatin treated cells along the thin lines are presented at different time points as indicated (first row). As a control, the actin structures after the “methanol only” treatment at 3% volume fraction are shown (second row). The step size was equal to 0.5 μm, while the number of images in the stack was 86.

The observations of fluorescent actin microfilaments in living cells showed that the actin filaments were assembled on the border of small blebs (Fig 5a). However, at higher nystatin concentrations, when the blebs became larger, the actin filaments were not observed on their borders anymore (Fig 5b). These results were also supported by the measurements carried out with the confocal microscopy (Fig 6). On the images taken 20 min and 60 min after the addition of 300 μmol/L of nystatin solution it could clearly be seen that the actin cytoskeleton collapsed. As is clear from Fig 6, the substantially changed structure of the actin microfilaments could still be detected in the lowest, attached part of the cells. Above this, some brighter spots, indicating aggregates of actin, were noticed, although they completely disappeared close to the apical surface of the “cell-vesicles” where no actin fibers or any other forms of actin organization could be discovered.

Control experiments without nystatin and with methanol volume fractions maintained from 1 to 10% volume fractions which correspond to nystatin concentrations between 100 and 1000 μmol/L showed no significant changes at methanol volume fraction equal to 1%. However, the rounding of the cell shapes was observed at higher methanol volume fractions (Fig 1, third row). The cell rounding was accompanied by the cell volume decrease which could be detected by the confocal fluorescence microscopy (Fig 6). At methanol volume fraction equal to 6%, an average continuous cell volume decrease of 20% was revealed within the first 10 min, afterwards the cell volume remained fairly constant for the rest of the observation time (comparable to the line in Fig 3b). At lower methanol volume fractions the decreases in cell volume were found to be smaller.

The cell viability studies after the nystatin application revealed significant concentration and time dependent effects. At nystatin concentration equal to 100 μmol/L the percentage of viable cells was significantly higher compared to higher concentrations. The fractions of viable cells with values between 60 and 35% (average = 47%, SD = 9%), normalized to untreated cells, were determined during the whole observation time after the nystatin application. At concentrations equal and higher than 150 μmol/L, the percentage of viable cells was lower than 30% (25% at 200 μmol/L and 10% at 300 μmol/L already 10 min after the application and it decreased to the values below 7% after 60 min of measuring time. In control, “methanol only” treated cells with the quantities of methanol corresponding to 100, 300 and 600 μmol/L nystatin suspension, comparable fractions of viable cells to the untreated cells were determined.

## Theory of Osmotic Effects

### Basic concept

The primary goal of this section is a basic understanding of the cell behavior after the formation of the nystatin transmembrane pores. The observed vesicle behavior has been found to be a consequence of the osmotic changes in the vesicle, induced by the presence of the size-discriminating pores [26,30,31]. They selectively change the permeability of the membrane and, consequently, the osmolarity inside the vesicle increases if higher concentrations of smaller molecules outside the vesicle with respect to its interiority initially exist. The osmotically induced flow of water into the vesicle causes the volume of the vesicle to increase.

The observed behavior in the cells can be regarded as an extension of the two-component system studied to gain an understanding of the tension-pore behavior caused by sugar gradients in the lipid vesicles [29]. The generalized equations determining the flows through the cell membrane due to the concentration gradient and the occurrence of the tension pore can be seen in S1 File.

### Permeability of nystatin pores for ions

Since nystatin forms cation and anion selective pores [9], the electric selectivity on the passage of charged ions, e.g., Na^+^, K^+^ and Cl^-^ ions inside and outside the cell, has to be considered as well. Their solute permeabilities can be conveniently written as

$$P_{i}=\alpha_{i}P_{R,i},$$ (1)

where α_*i*_ determine the influence of the electric selectivity and *P*_*R*,*i*_ are the permeabilities calculated using the expression given by Renkin [28]:

$${\text{P}}_{\text{R},\text{i}}={\text{D}}_{\text{i}}\frac{{\text{N}}_{\text{NP}}\pi {\text{r}}_{\text{NP}}^{2}}{\text{d}}\times {\left(\text{1}-\frac{{\text{r}}_{\text{i}}}{{\text{r}}_{\text{NP}}}\right)}^{\text{2}}\times \left(\text{1}-\text{2}.104\frac{{\text{r}}_{\text{i}}}{{\text{r}}_{\text{NP}}}+\text{2}.09{\left(\frac{{\text{r}}_{\text{i}}}{{\text{r}}_{\text{NP}}}\right)}^{\text{3}}-\text{0}.95{\left(\frac{{\text{r}}_{\text{i}}}{{\text{r}}_{\text{NP}}}\right)}^{\text{5}}\right)$$ (2)

with *D*_*i*_ the diffusion constants of ions, *N*_NP_ the number of nystatin pores, *d* the membrane thickness, and *r*_*i*_ and *r*_*NP*_ the effective radii of ions and nystatin pores.

### Conservation of the electrical charge and the total flow

If the solutes are charged, additional contributions of charged solutes, generated by the electrostatic potential, have to be considered as well. The flows of solutes through the nystatin pores have to be modified using an additional term [32]

$${\tilde{\Phi }}_{\text{NP},\text{i}}=\Phi_{\text{NP},\text{i}}-\frac{{\text{Z}}_{\text{i}}{\text{e}}_{0}}{{\text{k}}_{\text{B}}\text{T}}{\text{P}}_{\text{i}}\bar{{\text{c}}_{\text{i}}}\text{U},$$ (3)

where Φ_NP,i_ is the flow of solute molecules through the nystatin pores [Eq (10), S1 File], *Z*_i_ is the valence of the charged solutes, *e*_0_ is the elementary charge, *k*_*B*_ is the Boltzmann constant, *T* is the temperature, $\bar{{\text{c}}_{\text{i}}}$ is the mean solute concentration and *U* is the electric potential difference between the interior and the exterior of the cell, if a linear dependence of the potential on the position across the membrane is assumed. Similarly, the flows through the tension pore should be modified using

$${\tilde{\Phi }}_{\text{TP},\text{i}}=\Phi_{\text{TP},\text{i}}-\frac{{\text{Z}}_{\text{i}}{\text{e}}_{0}}{{\text{k}}_{\text{B}}\text{T}}\frac{{\text{D}}_{\text{i}}}{\text{d}}\pi {\text{R}}_{\text{TP}}^{2}\bar{{\text{c}}_{\text{i}}}\text{U},$$ (4)

where Φ_TP,i_ is the flow of solute molecules through the tension pore with the radius *R*_TP_ [Eq. (13), S1 File], *D*_*i*_ are the diffusion constants of the solutes and *d* is the membrane thickness. The electric field (*U/d*) at the tension pore was taken to be equal to the average electric field in the membrane.

In addition, to conserve the electric charge inside the cell, the sum of all the flows of the charged solute molecules has to be equal to zero at any time:

$$\sum_{\text{i}}{\text{Z}}_{\text{i}}({\tilde{\Phi }}_{\text{NP},\text{i}}+{\tilde{\Phi }}_{\text{TP},\text{i}})=0,$$ (5)

which leads to the expression for the potential *U*.

When the tension pore is open and the conservation of the electric charge is considered, the total volume flow can be written as the sum of the water flows through the nystatin pores, directly through the cell membrane and through the tension pore (Eqs (9), (11) and (12), S1 File)

$$\frac{\text{dV}}{\text{dt}}={\text{J}}_{\text{NP}}+{\text{J}}_{\text{B}}+{\text{J}}_{\text{TP}},$$ (6)

and the corresponding total flows of the solute molecules (Eqs (3) and (4)) as

$$\frac{\text{d}{\text{N}}_{\text{i}}}{\text{dt}}={\tilde{\Phi }}_{\text{NP},\text{i}}+{\tilde{\Phi }}_{\text{TP},\text{i}}.$$ (7)

In the model, we numerically solve Eqs (6) and (7) in order to calculate the time dependence of the cell volume and the radius of the tension pore (S1 File). The most abundant solutes inside and outside the cells were Na^+^, K^+^ and Cl^-^. Their initial cellular concentrations were taken to be 12, 139 and 4 mmol/L [33], and their initial concentrations outside the cell were 124, 4.8 and 129 mmol/L (Gibco, USA). The diffusion constants of Na^+^, K^+^ and Cl^-^ in water were taken to be 1.334, 1.957 and 2.032 × 10^−9^ m^2^/s [34], and their radii (*r*_*i*_), obtained using the Stokes-Einstein equation, were 0.184, 0.125 and 0.121 nm, respectively. Consequently, their reflection coefficients were estimated using a linear function, σ_*i*_ *= r*_*i*_/*r*_NP_, with *r*_NP_ being the radius of the nystatin pores [35]. The other solute molecules were taken to be either too large to pass the cell membrane through nystatin transmembrane pores or they were not taken into account due to their small concentrations. On average, these solute molecules were assumed to be without any electric charge. The total initial cellular concentration of all the solute molecules and the total initial concentration of all the solute molecules outside the cell were taken to be equal to the physiological concentration (300 mmol/l). The cell radius [*R*_c_ = (*A*_0_/4π)^1/2^, with *A*_0_ being the equilibrium membrane area] and its membrane thickness (*d*) were set to 18 μm and 5 nm. The values for the constants λ_c_, *k*_*A*_, Γ and *k*_c_ were set to 1.19×10^−2^ N/m, 0.354 N/m, 3.41×10^−11^ N and 86.8*k*_*B*_*T*, which correspond to the POPC membrane with 30% cholesterol. The permeability coefficient of the lipid bilayer (*l*_*PB*_) was set equal to 9 × 10^−13^ m^3^/(Ns) [36] and the hydraulic permeability for nystatin pores (*L*_*p*_) was estimated with *N*_NP_ × (π*r*_NP_^4^ /(8η*d*)), in accordance with the Hagen-Poisseuille law. The viscosity of the solutions was taken to be 0.9 × 10^−3^ Pas and the temperature was 310 K.

### Predictions of the model

After the formation of nystatin size-discriminating pores the concentration changes inside and outside the cell occur. They are a consequence of the increased permeability of the membrane for the electrolytes (Na^+^, K^+^, Cl^-^) and the persisting non-permeability with respect to proteins and other cellular macromolecules. Before the pore formation, the osmolarity of the solutions inside and outside the cell is equal; however, the concentration of the non-permeable cellular macromolecules is considerably (approximately 3 times) larger inside the cell. After the pore formation, the concentration of each permeable electrolyte ion tends to equalize, leading to an increase in the concentrations of Na^+^ and Cl^-^ and a reduction in the K^+^ concentration inside the cell.

**Fig 7:**
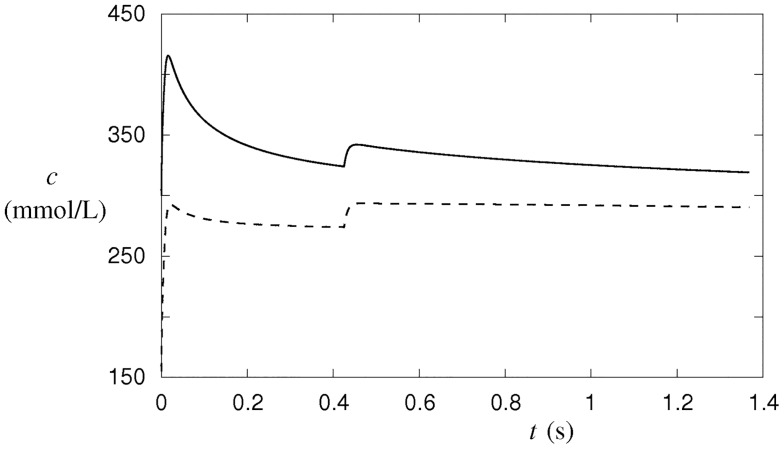
Concentration and osmolarity changes in the cell after the formation of 9 × 10^7^ nystatin pores with a 0.45-nm radius. The cellular osmolarity is depicted by full line and the sum of the cellular osmolarities (concentrations) of the most abundant ions by dashed line. The changes were predicted by the numerical procedure according to the model (S1 File).

When the number of nystatin pores with a radius of 4.5 × 10^−10^ m is taken to be equal to 9 × 10^7^, for example, two stages of ion exchange are predicted before the tension pore occurs (Fig 7). During the first stage, the total intracellular ion concentration increases rapidly. The second stage, which is terminated just before the tension-pore opening, is characterized by more moderate electrolyte exchanges. The rapid ion-exchange rate during the first stage is caused by the large transmembrane differences in the electrolyte concentrations, which is to a great extent dissipated before the second stage. The rate of exchange is moderated due to the requirement to conserve the total electric charge in the cell. However, it must be stressed that the number of cellular macromolecules remains unchanged during both stages, although their concentration is decreasing due to the increasing cell volume (Fig 7). As a consequence, the osmolarity of the cytosol increases from 300 mmol/L to its maximum value of 415 mmol/L during the first stage, followed by a slow asymptotic decrease to 300 mmol/L during the second stage (Fig 7). The cellular osmolarity is mainly decreased since the total concentration of the cellular macromolecules decreases.

**Fig 8:**
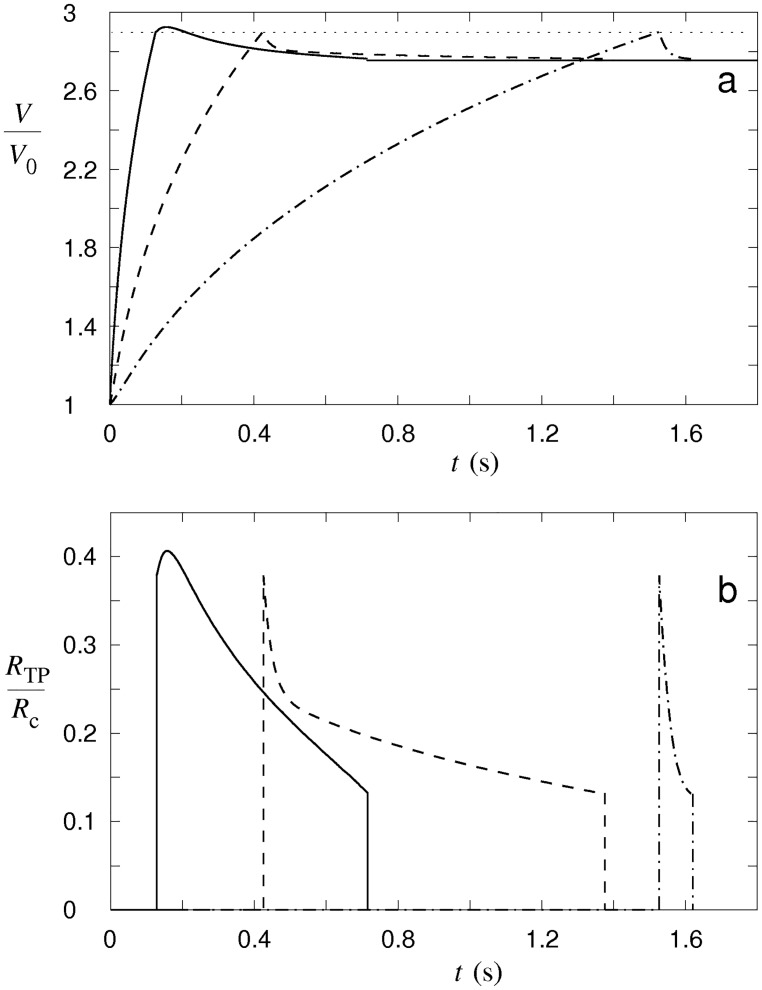
Predicted changes in the cell volume and in the tension-pore radius. The changes in the cell volume (a) and in the tension-pore radius (b) are shown for different numbers of nystatin pores 2.5 × 10^7^ (dash-dot line), 9 × 10^7^ (dashed line) and 3 × 10^8^ (full line), characteristic for type-I, type-II and type-III tension-pore behavior. The dotted line indicates the critical volume of the cell. The cell volume is normalized to its initial volume (*V*/*V*_0_) and the tension pore radius to the radius of the cell (*R*_TP_/*R*_c_). The radius of the nystatin pores is the same as in Fig 7. The numerical procedure is described in S1 File.

As a consequence of the concentration changes, the cellular osmotic pressure is built up, leading to a flow of water into the cell, which causes the volume of the cell to increase (Fig 8a). When the critical cell volume corresponding to the critical membrane tension is reached, the tension pore occurs. A portion of the cell content will be non-selectively extruded through the tension pore and a reduction of the cell volume will occur. The occurrence of the tension pore is thus essentially the only way that the cellular macromolecules can leave the cell and thereby reduce the cellular osmolarity. In contrast, the exchange of individual electrolytes through the tension pore can relatively fast be regarded as negligible since conservation of the electric charge becomes the limiting factor for the ion exchange after the tension-pore formation.

Similar to the behavior of tension pores in vesicles [29], three different cell-membrane responses are predicted by the model in an effort to compensate for the water flow into the cell (Fig 8b). At low numbers of nystatin pores, the water inflow, driven by the osmotic pressure, induces only a slowly increasing cell volume (Fig 8a). A tension pore whose radius is a maximum at its occurrence is predicted. Its radius then decreases quickly until the tension pore closes (type-I tension-pore, Fig 8b). Hence, at low numbers of nystatin pores, only a short opening of the membrane is sufficient for the cell volume to be reduced by losing a portion of the cellular content. If the number of nystatin pores increases, a long-lasting opening of the membrane occurs in order to prevent a further increase of the cell volume. The radius of this type of tension pore also decreases quickly after its occurrence; however, after a certain value is reached, the tension pore’s radius starts to decrease significantly more slowly and the tension pore stays open for a longer period of time (type-II tension-pore, Fig 8). Only in this way, a higher inflow of water driven by an increased osmotic pressure, which is caused by the intense net passage of ions into the cell, is compensated. For the largest numbers of nystatin pores a further increase in the tension pore’s radius after its occurrence is predicted. Thus, the tension pore’s radius exceeds its initial value (type-III tension-pore, Fig 8). The inflow of water at these high numbers of nystatin pores is so overwhelming that a tension pore with an increasing tension-pore radius is needed to stop a further cell-volume increase.

**Fig 9:**
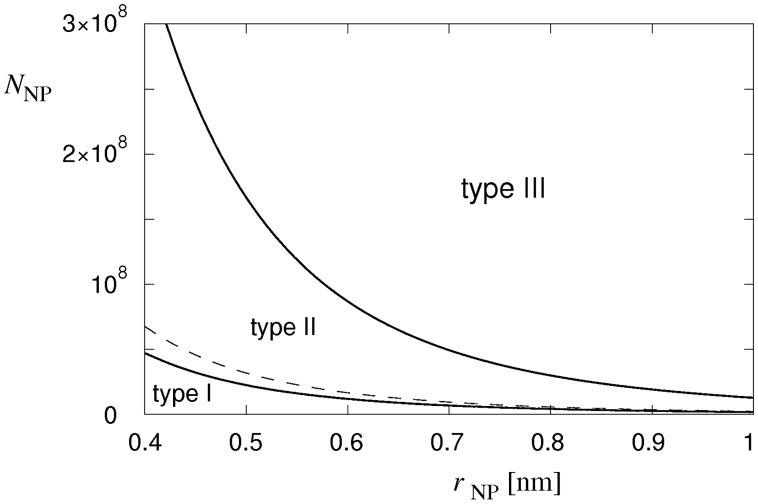
The characteristic types of tension-pore behavior. The three types of tension-pore behavior as a function of the radius and the number of nystatin pores (*r*_NP_ and *N*_NP_). The dashed line shows the border between the type-II and type-III tension-pore behavior for a ten-times-smaller lysis tension.

The phase diagram showing the borders between the type-I and the type-II cell behavior, and between the type-II and the type-III cell behavior, as a function of the nystatin pore numbers and the radius of the nystatin pores, is presented Fig 9. A wider range of nystatin pore radii is chosen in order to generalize the theoretical predictions. The border between the type-I and type-II tension-pore behavior is determined as the number of nystatin pores at which the opening time of the tension pore starts to become significantly larger (larger than 0.1 s). For a nystatin pore radius of 4.5 × 10^−10^ m, it is predicted to be at a nystatin pore number equal to 3.8 × 10^7^. Similarly, the border between the type-II and type-III tension-pore behavior is determined as the minimum number of nystatin pores at which the radius of the tension pore exceeds its initial value. For the same nystatin pore radius, it occurs at nystatin pore numbers higher than 2.4 × 10^8^. Note that this border is, for a ten-times-smaller lysis tension, shifted closely towards the border between the type-I and type-II tension-pore behavior that is, in contrast, not significantly affected by the changed lysis tension. The formation of nystatin pores can, for example, cause changes in the mechanical characteristics of the cell membrane, especially for high pore numbers. This effect could reduce the critical value of the line tension resulting in fast ruptures at smaller numbers of nystatin pores (Fig 9). This feature seems to be justified because the incidence of fast and slow ruptures was observed to be similar in our experiments. In general, an increase in the number of nystatin pores for a decreasing radius of nystatin pores for the same type of tension-pore behavior is predicted by the model.

The role of electric selectivity of nystatin pores was also examined within the model. The results show that if the coefficients α_*i*_ determining the ion selectivity of nystatin pores were set to 1, 3 and 1/3 for Na^+^, K^+^ and Cl^-^ ions [9,37], the borders between type-I and type-II tension-pore behavior as well as for type-II and type-III tension-pore behavior were shifted to higher numbers of nystatin pores. For a nystatin pore radius of 4.5 × 10^−10^ m, the nystatin pore number increased for 36% in the first and for 18% in the latter case at these extreme values of α_*i*_. For a reversed ionic selectivity [9], i.e., with coefficients α_*i*_ set to 1, 1/3 and 3 for Na^+^, K^+^ and Cl^-^ ions, these borders were shifted to lower numbers of nystatin pores. For the same radius of nystatin pores, the decreases of nystatin pore numbers were found to be equal to 15% in the first and 13% in the latter case.

## Discussion

**Fig 10:**
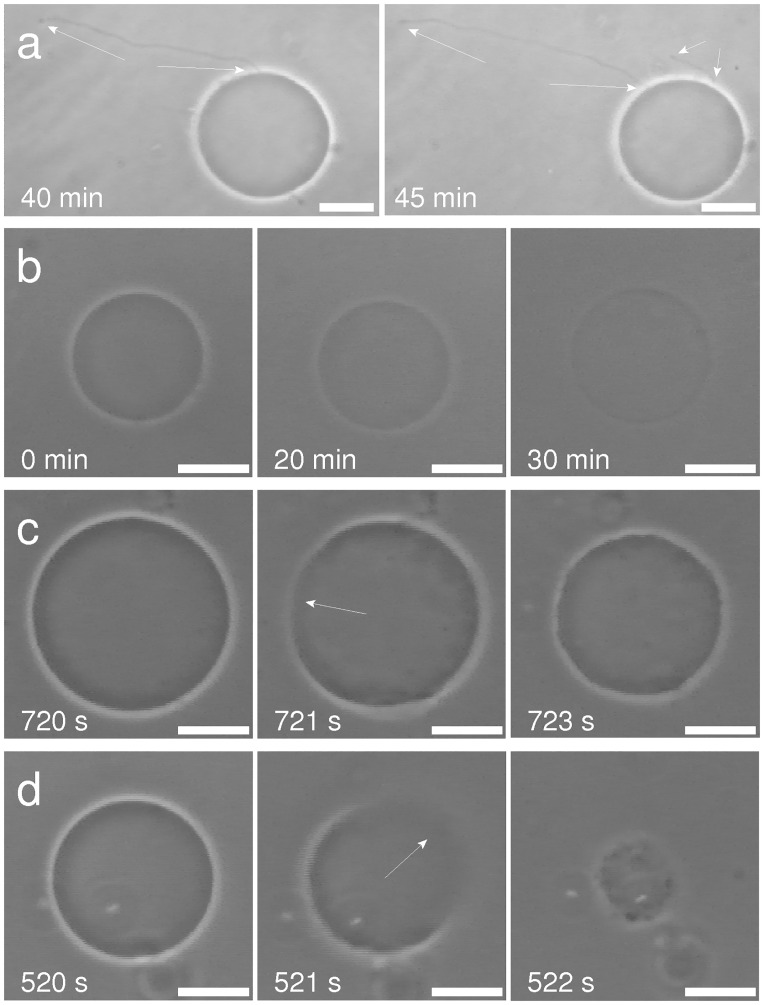
Characteristic behavior of GUVs at different nystatin concentrations. The GUVs show different behavior due to the pore-formation process in vesicles with smaller glucose molecules outside and larger sucrose molecules inside, as seen using phase-contrast microscopy [26]. Development of typical membrane protrusions (indicated by arrows) due to the nystatin binding to the outer membrane monolayer at low nystatin concentrations (a). Loss of vesicle contrast due to transient tension pores at intermediate nystatin concentrations (b). Slow (c) and fast (d) vesicle ruptures at high nystatin concentrations: GUVs before the rupture (left), during the rupture (middle, tension pore indicated by arrow), and immediately after the rupture (right) are shown. The times after the GUV transfer into the nystatin solution are depicted on the images. The bars represent 20 μm. For more detailed descriptions see Refs. [26] and [29].

The studies of the effects of the pore-forming agent nystatin on the lipid giant unilamellar vesicles (GUVs) showed a characteristic, concentration-dependent behavior (Fig 10). This behavior could be described by a theoretical model based on the pore-diffusion theory and the theory of osmotic lysis (S1 File, and Ref. [29]). A straightforward question arises as to what extent this behavior can be identified, interpreted and modelled in a more complex biological structure.

### Comparison of the cell and the lipid vesicle behavior

The results of the experiments in the cells show that in spite of a complex plasma-membrane structure and its attachment to the cytoskeleton, the response of the cell is driven by the same basic principles as in GUVs. The observed cellular effects, i.e., the detachment of cells, the formation of blebs, the occurrence of “cell-vesicles” and their ruptures (Section 3), are a consequence of the formation of size-selective nystatin pores in the membrane. Their formation leads to a greatly enhanced membrane permeability for ions that initially have a higher net concentration in the cell surroundings. Consequently, the intracellular ion concentration is raised after the capacity of the membrane active self-preservation mechanisms has been exceeded. In contrast, the number of large cellular macromolecules is fairly unchanged as their membrane permeabilities remain negligible. This leads to an increased osmotic pressure in the cell that induces an influx of water into the cell and accordingly, the cell volume increases. As a consequence, a tendency for an increased membrane area exists, and for a limited cell-membrane area the membrane’s lateral tension increases as well.

As is the case in GUVs, the cell behavior also depends strongly on the applied nystatin concentration, which is in close correlation with the number of nystatin pores [29]. At smaller nystatin concentrations, a relatively small number of nystatin pores is expected to induce only a slowly increasing volume of the cell that attains a more spherical shape. More spherical shapes can contain more volume for a given membrane area than the flatter shapes. Indeed, a thickening of the cell and a decrease of the cell footprint (adhered membrane area) are generally confirmed by the observations (Figs 1 and 6). The detachment, accompanied by more spherical shapes, with an increasing volume can be simulated by GUVs at lower filling factors [38]. It should be mentioned that, in contrast to the experiments with GUVs (Fig 10a), no membrane protrusions were observed for the lower nystatin concentrations (Fig 1a). Their appearance in the GUVs was interpreted as a consequence of an increased area of the outer membrane monolayer due to the binding of nystatin molecules onto it, which occurs as the first step of the pore-formation process [11,12,26]. It can be assumed that in cells, this increase of the outer cell membrane monolayer is compensated by the flattening of the invaginated membrane structures, e.g., the reduction in the number of caveolae, which in fact possess a larger inner membrane area [39,40].

The formation of blebs can also be understood as a consequence of an increased cell volume. Namely, if the pressure in the cell exceeds a certain value, which is large enough to disturb the binding of the membrane to the cytoskeleton, the membrane detaches from it, the hidden membrane area is stretched out and a bleb is formed [41–43], as seen in Figs 1 and 5. These blebs are small at nystatin concentrations around 100 μmol/L; however, at nystatin concentrations approaching 150 μmol/L, increased volumes and numbers of blebs, as well as their merging, are observed (Figs 1 and 5). This is caused by a more intensive water flow into the cells. The bleb formations could not be observed in our experiments with GUVs, since the vesicles have a homogenous membrane without proteins and no cytoskeleton.

The formation of one single bleb (“cell-vesicle”, Figs 1 and 2) that could be observed for the first time at 150 μmol/L nystatin concentrations is a consequence of a higher osmotic pressure due to larger numbers of nystatin pores, which is reflected in a significantly intensified water inflow. The cell-volume increase approaches a value that is approximately 190% higher than the initial value (Fig 3). This volume increase over a period of 1 hour is reflected in the “type A” cell behavior pattern (Fig 2b). At this stage, the diffusely disrupted binding of the membrane to the cytoskeleton is intensified due to a significant pressure increase. The volumes of blebs grow, the blebs merge and, finally, the binding of the membrane to the cytoskeleton is totally disrupted, resulting in the formation of a “cell-vesicle”. The cell membrane attains a spherical shape and the cell starts to behave like a strained GUV. The experiments on individual cells clearly show that the number of blebs decreases on an increasing volume of the cell. The tendency to lower number of blebs can be explained as the consequence of the need for more membrane material to form bleb surface at an increasing volume. Since this process should occur at the lowest energy possible, a lower number of blebs is preferable for their formation due to a smaller bleb area at a given volume. At a lower number of blebs the bending energy is also smaller due to a smaller average curvature of the membrane.

At nystatin concentrations above 250 μmol/L, where an early formation of “cell-vesicles” is observed, different types of tension pores are predicted by the model. The water inflow in these conditions is the greatest due to the largest numbers of nystatin pores. The cell membrane will be stretched to the point where the membrane tension reaches its critical value, which is followed by the appearance of the tension pore. Indeed, the experimentally determined maximum values of the “cell-vesicle” volume are reached at these concentrations (Fig 3), indicating that no hidden membrane area exists anymore. Slow or fast cell-membrane ruptures comparable to the irreversible ruptures in GUVs are observed (Figs 2 and 10), which is in accordance with the model predicting type-II and type-III cell behavior (Fig 8). However, the predicted type-I tension-pore behavior, indicating only a short, reversible opening of the membrane at the lower part of this concentration range (Fig 8), which has already been observed in GUVs, could not be detected directly. First of all, its potential detection is experimentally difficult due to the lack of any contrast between the inner and the outer cell solution. In addition, the immobilization of cells and their collisions with their neighbors did not allow the detection of small movements, characteristic for the appearance of reversible tension pores in GUVs. We could deduce them only indirectly from the reduction in the fluorescence signal in the “cell-vesicle” (Fig 4), which could result from the extrusion of the fluorescent dye calcein out of the cell through the reversible tension pores. In fact, the steady cell volume in the “type B” cell behavior pattern (Fig 3b), which is detected after the maximum cell volume has been reached, can be explained as a consequence of short, reversible openings of the membrane (type-I tension-pore behavior). On the other hand, the decreases of the cell volume, which are detected predominantly at highest nystatin concentrations after their maximum has been reached and are reflected in the “type C” behavior pattern (Fig 3b), are explained as a consequence of irreversible slow cell-membrane ruptures (“type-II tension-pore behavior”). Similar cell changes were observed also in different cell lines in a hypotonic buffer [44].

The cell behavior and the appearance of tension pores are determined, as already mentioned, by an influx of water molecules into the cell, resulting in an increased membrane tension. The tension pore closes when the membrane's elastic energy with an open tension pore is equal to the membrane's stretching energy with a closed tension pore. This behavior is strongly dependent on the mechanical properties of the membrane, e.g. the membrane stretching constant (*k*_*A*_), the line tension (Γ) and the critical membrane tension (λ_c_). The model predicts that an increase in *k*_*A*_ generally has an opposite effect as an increase in Γ or λ_c_. For example, an increase in *k*_*A*_ makes the membrane less elastic and accordingly, more difficult to stretch. A more rigid membrane results in smaller volume increases and critical tension is reached earlier. Accordingly, the tension pore is formed at lower numbers of nystatin pores. In contrary, an increase in Γ causes a greater tendency towards tension pore contraction, thus the tension pores are smaller and close faster. A higher number of nystatin pores is also needed to open the tension pore in a membrane with a higher λ_c_.

Finally, the control experiments undertaken solely with methanol which was used as a solvent for nystatin should be discussed. They showed nontrivial effects of methanol at higher methanol volume fractions which correspond to the experiments with nystatin concentrations higher than 100 μmol/L. The rounding of the cells (Figs 1 and 6) and their volume decrease in the first 10 min which stabilized at a value of around 80% of the initial ones (Fig 3b) were observed after the use of methanol in volume fractions corresponding to the maximal nystatin concentration (600 μmol/L). These effects can be explained by an increase of the osmotic pressure outside the cells due to an increased concentration caused by the methanol molecules with a smaller permeability through the cell membrane compared to the water molecules. Consequently, a water outflow and a diminishment of the cell volume occur. The observations do not indicate a membrane change by itself in experiments with nystatin concentrations equal to or below 600 μmol/L. This is in agreement with the results of the viability tests which show comparable results in “methanol only” treated cells to the untreated cells and significantly higher fractions of viable cells compared to the nystatin treated cells (Section 3). Thus, the use of methanol as a solvent potentially affects only the time dependence of the observed cell volume changes.

### Adaption of the lipid vesicle model for the description of the cell behavior

The model that was able to give a basic understanding of the tension-pore behavior in the lipid vesicles [29] had to be “upgraded” to understand the experimental results in the cells. The description for the GUVs is namely based only on a two-component model with glucose (higher permeability) outside and sucrose (lower permeability) inside the vesicle. In the cell model, however, the multicomponent situation with several ions and large molecules has to be encountered. In our model, only the most abundant ions (Na^+^, K^+^, Cl^-^) and the large molecules with concentrations over 5 μmol/L inside or outside the cell were considered. The limitation to these ions and large molecules is reasonable since the osmolarities of these components inside or outside the cell were almost equal before the onset of the nystatin pores. In this sense, the cell model resembles, in spite of a much more complex cell structure, the two-component glucose-sucrose model of the GUVs, with a net concentration of ions with higher permeabilities outside the cell and the cellular macromolecules with low permeabilities inside the cell [29,30]. Secondly, the flows of electrically charged ions and the electrical neutrality of the cell also had to be considered. The cell was considered to be neutral at the beginning and this constraint of zero electric charge inside the cell was taken into account also after the pore formation, when the flows of charged solute ions across the membrane occurred. As a consequence, the electric forces were induced by the electric charges in the system in addition to the osmotic forces. As a simplification, the cellular macromolecules and macromolecular ensembles that contribute to the cellular osmolarity were assumed to be electrically neutral on average. In addition, the numerical simulations revealed only a limited role of the electric selectivity of nystatin pores for Na^+^, K^+^ and Cl^-^ ions in the “cell-vesicle” behavior.

It should be noted that the temporal development of the nystatin transmembrane pores, i.e., the changes in their radii and surface densities, were not taken into account, similar to the case of the lipid vesicle model. Although these changes cannot be excluded, this approximation is reasonable, since all these aspects turned out to be less important for a basic understanding of the observed effects. Similarly, as a first approximation, no viscosity of the cell membrane is taken into account [45]. Furthermore, a linear interrelation between the inner cell pressure and the cell-volume increase is assumed, which is essentially expected due to a gradual detachment of the membrane from the substrate and the cytoskeleton. Indeed, essentially the same basic characteristics were obtained from the model simulations using small deviations from linearity.

In spite of these model limitations, our main goals, the basic understanding of the cell behavior induced by nystatin and the correlation with the corresponding responses in the GUVs, could be achieved. The model predicts comparable effects to those in GUVs.

### Biological aspects

The formation of nystatin pores in the cellular membrane leads, as already mentioned, to a considerably increased permeability and consequently to a significant flow of ions through the cell membrane. Normally, the cells should solve the ionic imbalance by active transport. However, the morphological changes observed within one hour of our observation time show that the damage induced by the nystatin at concentrations at least above 100 μmol/L was too large. The large increase of the cellular volume led to the formation of larger blebs and “cell-vesicles”, typical predecessors to the ruptures of the plasma membrane (Fig 2), which are a typical feature of cell necrosis [46]. The observed behavior is in alignment with the cell viability studies which revealed that the percentage of viable cells was significantly higher after exposure to 100 μmol/L nystatin solution than after exposure to 150, 200 or 300 μmol/L nystatin solutions. In the first case, the fractions of viable cells was namely between 60 and 35% at the end of the measuring time, while at higher concentrations the values were lower than 30% already 10 min after the nystatin application and decreased to values below 7% in the rest of the observation time.

The comparison between the experiments and the theoretical results shows that the cell tries to preserve its membrane integrity for an increasing cell volume using different morphological changes (Fig 1). The results indicate that in CHO cells at small volume changes the flattening of the invaginated membrane structures occurs. At increased cell volumes the membrane detaches from the cytoskeleton and smaller or larger (over approximately 70% volume increase) blebs occur (Fig 5). Both phenomena, flattening of the membrane and bleb formation, are in accordance with the recent findings in the literature [39,40,42,43]. From the occurrence of more spherical cell-shape transformations it can be concluded that the energy necessary for the detachment of the membrane from the cytoskeleton is comparable to the energy necessary for the detachment of the membrane from the substrate. Namely, more spherical cell shapes can gradually evolve only in this case.

The formation of “cell-vesicles” at cell-volume increases higher than approximately 140% can be, due to their larger cell volume to membrane area ratios, interpreted as the last way to keep the membrane tension below the critical level in order to preserve the membrane’s mechanical integrity. However, the appearance of the tension pores at an approximate 190% cell-volume increase (Fig 2) indicates that the membrane reservoir has come to its limit. The role of the cell membrane in separating the cell interior from the surroundings at the highest nystatin concentrations is comparable in “cell-vesicles” and GUVs, which is underlined by a good overlap of the theoretical and experimental results. It also indicates that the cell membrane’s mechanical characteristics are comparable with those of the GUVs, despite the apparent differences in their structural complexity, and that they are still largely preserved. It should also be noted that a fairly good overlapping of the nystatin concentration ranges between the experiments with the GUVs and the “cell-vesicles”, in spite of a more complex cell structure, has been found.

It should be mentioned that the maximum cell volumes as well as the cell volume increases in a short time after the nystatin treatment, normalized to the volumes before the treatment, decrease with an increased cell volume at all measured nystatin concentrations except at the lowest one (Fig 3a and 3d). The average slope of linear fits is equal to– 1.25 × 10^−4^ /μm^3^. The cell volume decrease is not found at 150 μmol/L nystatin concentration presumably due to a higher scatter of the data. However, it should be kept in mind that also the cell membrane area, i.e., its shape, plays an important role determining the effects of a pore-forming substance. Nevertheless, a linear dependence is found if the relative cell volume increases ((*V*_1_-*V(0)*)/*V(0)*) are plotted as a function of the ratio between the cell areas (*A*), which are determined from the spherical shape at maximum cell volume (*V*_max_), and the volumes before the treatment (*A*/*V(0)*). An average slope with a value equal to 2.0 μm was obtained for linear fits at measured nystatin concentrations. This dependency demonstrates that the flow rates through the membrane and thus, the cell volume increases are dependent on the number of the nystatin pores which is proportional to the membrane area. Furthermore, a rough estimation based on the determined cell volume increases in a short time after the nystatin application and the theoretical predictions (Section 4) shows that an average number of nystatin pores in order of 10^4^ is needed to induce such a cell volume increase. This shows a similarity to the results obtained in phospholipid vesicles [29].

Our experimental results also show that “cell-vesicles” and even larger blebs possess no actin fibers or any other forms of actin organization, especially in the upper part of the cells (Figs 5 and 6), that provide mechanical resistance. This is explained by the actin-degradation process due to the proteolytic enzyme activation as a part of the apoptosis or necrosis of the cells exposed to overwhelming osmotic stress [47,48], which was demonstrated also by the cell viability studies (Section 3).

At the end, it has to be emphasized that the cell is a complex system and that many sometimes interconnected signaling pathways and complementary homeostatic mechanisms, e.g. active transport of ions, can be activated upon the cell exposure to environmental stress. Thus, the incidence of the osmotic effects induced by the appearance of nystatin transmembrane pores is only one of the many factors influencing the cell integrity. However, our approach is justified at least at higher nystatin concentrations where the total depletion of ATP occurs due to the onset of necrotic effects.

## Conclusions

In previous studies, a characteristic, concentration-dependent behavior was revealed after the application of the pore-forming agent nystatin to lipid giant unilamellar vesicles (GUVs) as a cell-sized model membrane. In this work we focus on the effects induced by nystatin on Chinese hamster ovary epithelial cells and correlate them to those obtained in GUVs.

It was found that the application of the pore-forming agent nystatin induces a volume increase in the model membranes as well as in mammalian cells. This occurs due to the size-selective properties of the formed nystatin transmembrane pores that drastically change the permeabilities of the smaller solute molecules for an almost unchanged permeability of the larger ones.

A theoretical model based on the pore-diffusion theory and the theory of osmotic lysis which describes the occurrence of vesicle ruptures in GUVs was extended to take into consideration the flows of charged ions and the conservation of the electric charge density in the cells. The extended model predicts a tension-pore behavior comparable to that found in experiments with GUVs.

Although the basic principles are the same in GUVs and in the cells, the characteristic cell behavior revealed a diverse set of cell responses, primarily due to the reservoir of the hidden membrane on one side and the attachment of the cell membrane to the cytoskeleton on the other. The formation of blebs of gradually increasing dimensions, which then finally merge into a single bleb, referred to as a “cell-vesicle”, was seen only in the cells. However, the slow and fast cell ruptures that occur at higher nystatin concentrations corresponding to cell-volume increases as high as 190% closely resemble the irreversible ruptures found in experiments with GUVs [29].

Based on the previous studies on the model membranes, a theoretical model and experimental results are presented as an effort to elucidate the basic osmotic effects induced by the pore-forming agent nystatin in the cell. Nevertheless, future theoretical studies including temporal development of nystatin pores and experimental efforts including the measurements of fluorescently marked nystatin and ion sensors which exceed the scope of present study will accomplish the understanding of the activity of the pore-forming agent nystatin in more detail.

## Supporting Information

S1 File Numerical and experimental details. Supplementary File provides the description of the flows through the cell membrane, the tension pore formation, the pressure difference caused by the line tension, and the details concerning numerical solutions of the equations. A more detail description of the experimental conditions and the results on the giant unilamellar vesicles are also provided.
(DOCX)

S1 Video Slow rupture. A slow cell rupture with a significant leakage of the vesicle content and a substantial decrease of the “cell-vesicle” radius.
(AVI)

S2 Video Fast rupture. A fast cell rupture leading to a collapse of the “cell-vesicle”, accompanied by a total fragmentation of its membrane.
(AVI)
