# Supplementary material for: Osmotic Effects Induced by Pore-Forming Agent Nystatin: From Lipid Vesicles to the Cell
Source: PLoS One. 2016 Oct 27;11(10):e0165098. doi: 10.1371/journal.pone.0165098 (PMC5082891; doi:10.1371/journal.pone.0165098)
Supplement: S1 File — Supplementary File provides the description of the flows through the cell membrane, the tension pore formation, the pressure difference caused by the line tension, and the details concerning numerical solutions of the equations. A more detail description of the experimental conditions and the results on the giant unilamellar vesicles are also provided. (DOCX) [file pone.0165098.s001.docx]

**S1 File**

Flows through the cell membrane and the occurrence of the tension pore

The theoretical model for the lipid vesicles [1] can be generalized to describe the tension-pore behavior in the cells.

Flows through the cell membrane caused by the concentration gradient

The osmotic pressure difference between the interior and the exterior of the vesicle or cell is given by the equation

$\Delta\pi=k_{B}T\sum_{i} \left(\frac{N_{i}}{V}-c_{i}\right)$, (8)

where *i* refers to the different types of molecules that contribute to the osmosis. *k_B_* is the Boltzmann constant, *T* is the temperature, *N_i_* denotes the numbers of molecules in the cell with volume *V*, and *c_i_* is the number density of the corresponding molecules outside the closed membrane.

In order to determine the number of molecules and their number densities in the cell, three types of flows through the membrane must be considered: the flows of solutes (different molecules and ions), the flow of water that occurs through the nystatin transmembrane pores, and the flow of water that occurs directly through the cell membrane. The flow of solutes through the membrane (Φ*_NP,i_*) can occur only through the nystatin pores and is regulated by the size-discriminating properties of the pores. The flow of water through the nystatin pores (*J_NP_*) is interrelated with the flow of the solutes. Both the water flow through the nystatin pores and the flow of solutes depend on the difference in the hydrostatic pressures inside and outside the cell (Δ*p*), caused by the tensed membrane and the differences in the osmotic pressure, caused by the differences in the molecular number densities between the inner and the outer solutions (Δ*c_i_ = N_i_/V-c_i_*) [2]. The water flow through the nystatin pores can be expressed as

$J_{NP}=-L_{p}(\Delta p-k_{B}T\sum_{i} \sigma_{i}\Delta c_{i})$ , (9)

and the flow of solutes as

$\Phi_{NP,i}=(1-\sigma_{i})\overline{c_{i}}J_{NP}-P_{i}\Delta c_{i}$, (10)

where *L*_p_ is the hydraulic permeability for the pores, σ*_i_* and $\overline{c_{i}}$ are the reflection coefficients and the mean solute concentrations ((*N_i_/V+c_i_*)/2), and *P*_i_ are the solute permeabilities [Eq. (1)].

The water flow directly through the cell membrane can be expressed in an analogous way as

$J_{B}=-Al_{B}(\Delta p-k_{B}T\sum_{i} \Delta c_{i})$, (11)

where *A* is the membrane area and *l*_B_ is the permeability coefficient of the lipid bilayer with respect to water.

Occurrence of the tension pore

The volume of the closed membrane increases due to the net inflow of molecules, particularly water. As a consequence, the membrane’s lateral tension increases. A linear increase of the pressure in the cell with respect to the cell volume is assumed. When the cell-membrane tension exceeds the critical membrane tension (λ_c_), a tension pore occurs [3–5]. This offers an additional possibility for the passage of water and solutes out of the cell.

The volume flow through the tension pore can be described by the equation [6]

$J_{TP}=\frac{R_{TP}^{3}\Delta p}{\text{3η}}$ , (12)

where *R*_TP_ is the radius of the tension pore, η is the viscosity of the solution and Δ*p* is the hydrostatic pressure difference [Eq. (18)].

The flows of solute molecules through the tension pore are proportional to the volume flow. Thus, the corresponding flows can be written as

$\Phi_{TP,i}=\frac{N_{i}}{V}J_{TP}$ . (13)

The tension pore closes when the cell loses a large amount of its content. It is assumed that the tension pore closes when the elastic energy of the fluctuating membrane with an open tension pore becomes equal to the membrane’s stretching energy with a closed tension pore [Eq. (16)].

Pressure difference caused by the line tension

When the membrane tension reaches its critical value (λ_c_), a tension pore occurs on the cell vesicle membrane. Consequently, the area of the membrane and its lateral tension decrease significantly. The membrane area (*A*) is determined by the radii of the tension pore (*R*_TP_) and of the vesicle (*R*_v_)

$A=4\pi\text{R}_{v}^{2}-\pi\text{2R}_{v}\left(R_{v}-\sqrt{R_{v}^{2}-R_{TP}^{2}}\right)+\Delta A$, (14)

where Δ*A* is the total excess area due to the membrane undulations. It can be estimated by [7]

$\Delta A=A_{0}\frac{k_{B}T}{8\pi\text{k}_{\text{c}}}\ln\frac{(R_{v}/d)^{2}+\lambda R_{v}^{2}/k_{c}}{1+\lambda R_{v}^{2}/k_{c}}$, (15)

where *A*_0_ is the equilibrium membrane area, *k*_c_ is the membrane bending constant, λ_c_ is the membrane tension and *d* is the membrane thickness.

The area of the membrane is slightly larger than the equilibrium area difference due to the tendency of the line tension to reduce the tension pore. The mechanical energy of the membrane (*W*) is determined by the sum of the membrane’s stretching energy and the energy of the rim of the tension pore [8]

$W=\frac{k_{A}}{2A_{0}}\left(A-A_{0}\right)^{2}+\Gamma\times2R_{TP}\pi$, (16)

where *k*_A_ is the membrane stretching constant and Γ is the line tension. The radius of the tension pore corresponds to the minimum of *W* at a given *R_v_.* At equilibrium the tension-pore radius is determined by ∂*W*/∂*R*_TP_ = 0 which yields

$\frac{k_{A}}{A_{0}}(A-A_{0})\left(\frac{-2\pi R_{v}R_{TP}}{\sqrt{R_{v}^{2}-R_{TP}^{2}}}+\frac{\partial\Delta A}{\partial R_{TP}}\right)+2\pi\Gamma=0$. (17)

In order to express the pressure difference between the pressure in the cell and its surroundings (Δ*p*) the lateral tension in the membrane [*k_A_*(*A - A*_0_)/*A*_0_] can be expressed by this difference using Laplace equation, *k_A_*(*A - A*_0_)/*A*_0_ = *R_v_*Δ*p*/2. Neglecting *R*_TP_ with respect to *R_v_* and omitting the changes in the membrane excess area due to the changes in *R_v_*, the pressure difference can be approximated by the expression

$\Delta p=\frac{\text{2Γ}}{R_{v}R_{TP}}$ . (18)

It can be seen that the pressure in the vesicle is larger than the pressure in the surroundings due to the line tension. The pressure difference increases on decreasing tension pore radius due to the larger changes in the energy of the rim of the tension pore than in the membrane’s stretching energy.

Numerical solutions of the equations

The time dependencies of the cell volumes and the tension pore radii (Fig 7) as well as the dependencies of the cellular densities of the solute molecules (Fig 8) are solved numerically. The whole process is divided in two stages. The first stage describes the volume increase until the onset of the tension pore whereas the second stage describes the behavior of the tension pore. Each stage is subdivided into steps.

In each step of the first stage the pressure inside the cell is firstly calculated according to the assumption of its linear increase from the zero value at the initial cell volume to the critical pressure. After the pressure inside the cell is calculated the volume flow through the nystatin pores according to Eq. (9) is determined. Afterwards the membrane potential is determined [Eq. (5)], and the flows of the ions and the water flow directly through the cell membrane are calculated [Eq. (3) and Eq. (11)]. Finally, the new values of the parameters are calculated. These steps are iteratively repeated until the critical lateral tension is reached. The critical pressure is determined according to the Laplace equation (Δ*p*_c_ = 2λ_c_/*R_v_*). The largest possible volume of the cell before the tension pore formation is given by equation 4π*R*_c_^3^(1+ λ_c_/*k_A_*)^3/2^/3 where the *R*_c_ is equal to the radius of a sphere that has the equilibrium area of the cell membrane *A*_0_=4π*R*_c_^2^.

The second stage describing the tension pore behavior is also subdivided into steps. Firstly, the radius of the cell vesicle, calculated from its volume, *R_v_* = (3*V*/4π)^1/3^, and the radius of the tension pore, corresponding to the minimum of the elastic energy [expression (16)], are determined. Afterwards, the pressure inside the cell is calculated [Eq. (18)] and the volume flows through the nystatin pores and tension pore are calculated [Eq. (9) and Eq. (12)]. Similarly to the first stage, the membrane potential is determined [Eq. (5)], and the flows of the ions through the nystatin pores are calculated [Eq. (4)]. At the end the total flow through the membrane is determined using Eq. (6) and Eq. (7). The differential equations for the flows are integrated until the mechanical energy of the cell with the tension pore equals the mechanical energy of the cell with the closed tension pore [Eq. (16)].

Experiments using the GUVs

A more detailed description of the experimental conditions and the results on the giant unilamellar vesicles (GUVs) is presented in this section, however, a complete description can be found in the text and the supplementary video material of Refs. [9] and [1].

Materials and methods:

The GUVs were prepared from 1-palmitoyl-2-oleoyl-sn-glycero-3-phosphocholine (POPC) (Avanti Polar Lipids, USA) by electroformation according to the modified method of Angelova et. al. [10]. The formed vesicles containing 0.2-mol/l sucrose solution were kept at room temperature and used within three days of preparation. A two-compartment cell was used for the GUV measurements [9]. The first compartment contained the vesicles in a 0.2-mol/l glucose solution. Unilamellar, nearly spherical vesicles with diameters of 40 ± 20 μm were transferred by the micropipette in groups of 1 to 5 vesicles into the measuring compartment which was filled with 0.2-mol/l glucose solution and nystatin in a desired concentration. The GUVs were observed by optical microscopy using the phase-contrast technique (IMT-2, Olympus, Japan; objective LWD DM 60X, NA = 0.55). The images were acquired using a CCD camera (C5985, Hamamatsu, Japan). The obtained images of GUVs were analyzed on the qualitative level by a continuous observation of phase-contrast images by three independent observers. The quantitative analysis of GUVsˇ leakage was accomplished assessing the brightness profile of the halo intensity across the vesicle membrane which is dependent on the changing sucrose-glucose ratio inside the vesicle [9].

Effects of nystatin on GUVs

The results on GUVs demonstrate a significant dependence of characteristic responses on the nystatin concentration [9]. As the nystatin concentration increased over a certain threshold value of around 150 μM, various membrane formations were detected. Their shapes and sizes were quite diverse, ranging from tether like protrusions to groups of minute spheres (Fig 10a). At intermediate concentrations, i.e. in the range between 250 and 400 μM nystatin concentration, the contrast loss of GUVs was observed (Fig 10b) due to several membrane openings (transient pores). At concentrations above 400 μM vesicle ruptures, denoting slow vesicle tension pores or quick disintegration of the entire vesicle were detected (Fig 10c and Fig 10d). Slow ruptures were characterized by the opening of the vesicle membrane for up to several seconds and a significant part of vesicle content leaked out (Fig 10c). If the membrane resealed, the diameter of the vesicle was found to be considerably reduced. Fast ruptures or explosions were characterized by a sudden opening of the vesicle membrane and the vesicle, which seemed intact, just a fraction of a second ago, simply disappeared (Fig 10d).

Similarly to the cells, the observed behavior can be regarded as a consequence of an osmotic stress in the vesicles after the formation of size-discriminating nystatin pores [11]. Glucose molecules with a smaller effective radius (like the ions) experience, as a consequence, a higher membrane permeability than the sucrose molecules (like large cellular macromolecules in the cells) [12,13]. Hence, the osmolarity of the solution inside the vesicle, which is initially equal to that of the glucose solution outside the vesicle, gradually increases and an influx of water molecules into the vesicle results in an increased membrane tension [14,15]. The membrane burst (tension pore) occurs when the membrane tension reaches its critical value (Fig 10c).

**References**

1. Kristanc L, Božič B, Gomišček G. The role of sterols in the lipid vesicle response induced by the pore-forming agent nystatin. Biochim Biophys Acta. 2014; 1838: 2635–2645.
2. Katchalsky A, Curran PF. Nonequilibrium thermodynamics in biophysics. Cambridge, MA: Harvard University Press; 1965.
3. Bloom M, Evans E, Mouritsen OG. Physical properties of the fluid-bilayer component of cell membranes: a perspective. Q Rev Biophys. 1991; 24: 293–397.
4. Evans E, Heinrich V, Ludwig F, Rawicz W. Dynamic tension spectroscopy and strength of biomembranes. Biophys J. 2003; 85: 2342–2350.
5. Hsueh YW, Chen MT, Patty PJ, Code C, Cheng J, Frisken BJ, et al. Ergosterol in POPC membranes: physical properties and comparison with structurally similar sterols. Biophys J. 2007; 92: 1606–1615.
6. Happel J, Brenner H. Low Reynolds number hydrodinamics. The Hague, The Netherlands: Martinus Nijhoff Publishers; 1983.
7. Evans E, Rawicz W, Smith BA. Concluding remarks, Back to the future: mechanics and thermodynamics of lipid biomembranes. Faraday Discuss. 2013; 161: 591–611.
8. Koslov MM, Markin VS. A theory of osmotic lysis of lipid vesicles. J Theor Biol. 1984; 109: 17–39.
9. Kristanc L, Svetina S, Gomišček G. Effects of the pore-forming agent nystatin on giant phospholipid vesicles. Biochim Biophys Acta. 2012; 1818: 636–644.
10. Angelova MI, Soleau S, Meleard P, Faucon F, Bothorel P. Preparation of giant vesicles by external AC electric fields: kinetics and applications. Prog Colloid Polym Sci. 1992; 89: 127–131.
11. Kleinberg ME, Finkelstein A. Single-length and double-length channels formed by nystatin in lipid bilayer membranes. J Membr Biol. 1984; 80: 257–269.
12. Schultz SG, Solomon AK. Determination of the effective hydrodynamic radii of small molecules by viscometry. J Gen Physiol. 1961; 44: 1189–1199.
13. Wood RE, Wirth FP Jr., Morgan HE. Glucose permeability of lipid bilayer membranes. Biochim Biophys Acta. 1968; 163: 171–178.
14. Mally M, Majhenc J, Svetina S, Žekš B. Mechanisms of equinatoxin II-induced transport through the membrane of a giant phospholipid vesicle. Biophys J. 2002; 83: 944–953.
15. Mally M, Majhenc J, Svetina S, Žekš B. The response of giant phospholipid vesicles to pore-forming peptide melittin. Biochim Biophys Acta. 2007; 1768: 1179–1189.
